# Simultaneous Determination of Norfloxacin and Tinidazole Binary Mixture by Difference Spectroscopy

**Published:** 2011-06

**Authors:** Noura H. Abou-Taleb, Dina T. El-Sherbiny, Dalia R. El-Wasseef, Mohamed A. Abu El-Enin, Saadia M. El-Ashry

**Affiliations:** *Medicinal Chemistry Department, Faculty of Pharmacy, Mansoura University, Mansoura, Egypt*

**Keywords:** difference spectroscopy, norfloxacin, tinidazole

## Abstract

A simple and rapid difference spectroscopic method was developed for the simultaneous determination of binary mixture of norfloxacin (NF) and tinidazole (TZ) without prior separation. The proposed method depends upon measuring the absorbance of NF at 291.6 nm which is the zero crossing point on the difference spectra of TZ in 0.1 N NaOH *vs.* 0.1 N HCl. Similarly, the absorbance of TZ was measured at 344.4 nm which is the zero crossing point on the difference spectra of NF. Beer’s law was obeyed in the concentration range of 2-20 and 5-50 μg/mL for NF and TZ, respectively. The lower limits of detection (LOD) of NF and TZ are 0.23 and 0.36 μg/mL, respectively, while the lower limits of quantification (LOQ) of NF and TZ were 0.70 and 1.08 μg/mL, respectively. The precision of the method was satisfactory; the maximum value of relative standard deviations did not exceed 1.5% (n=10). The accuracy, expressed as recovery is between 98.25 and 101.8% with relative error of 0.29 and 0.23 for NF and TZ, respectively. The proposed method was successfully applied for the determination of both drugs in bulk powder, laboratory prepared mixture and commercial dosage forms such as tablets without interference from the commonly encountered excipients and additives. The results obtained are in good agreement with those obtained by the reference methods.

## INTRODUCTION

**Figure 1:**
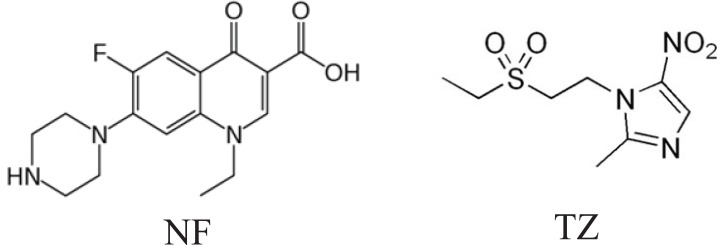
Structural formulae of the norfloxacin (NF) and tinidazole (TZ).

Norfloxacin (NF), [1-ethyl-6-fluoro-1, 4-dihydro-4-oxo-7-(piperazin-1-yl) quinoline-3-carboxylic acid], is a fluoroquinolone carboxylic acid derivative used as broad-spectrum antibiotic (Fig. 1). The mode of action of NF depends on blocking of bacterial DNA replication through inhibition of the bacterial DNA gyrase enzyme. It is used for treatment of uncomplicated urinary tract infections including cystitis and prostatitis. NF is the subject of a monograph in each of British Pharmacopoeia, BP (1) and the United States Pharmacopoeia, USP (2). The BP and USP recommended non aqueous titration for the raw material and HPLC (high performance liquid chromatography) methods for tablets. Because of the therapeutic importance of NF, numerous analytical methods have been developed for its determination in bulk, pharmaceutical formulations and/or biological fluids. Spectrophotometric technique is the most widely used in pharmaceutical analysis (3-7). Other analytical methods have been used such as spectrofluorimetry (8), HPLC (9-11), electrochemical analysis (12, 13), luminescence (14-18), and capillary electrophoresis (17, 18).

Tinidazole (TZ), [1-(2-(ethylsulfonyl) ethyl)-2-methyl-5-nitroimidazole], is an effective antiprotozoal and antibacterial agent (Fig. 1). It is used for treatment of amoebiasis, giardiasis and trichomoniasis. TZ is the subject of monograph in each of the BP (1) and the USP (2). The BP and USP recommended non aqueous titration for determination of TZ. There are several reports on the determination of TZ, both in formulations and biological fluids, *viz*: spectrophotometry (19-25), HPLC (26-30), and electrochemical analysis (31-33).

The combination of NF and TZ is commercially available in tablet form to control gastrointestinal infections caused by bacterial or amoebic infection, prostatitis and urinary tract infections due to susceptible uropathogens. Both drugs were simultaneously determined by spectrophotometry (34-36), HPLC (37), electrochemical analysis (38), and capillary electrophoresis (39).

Difference spectrophotometry has proved particularly useful in the determination of drugs by eliminating specific interference from degradation products and co-formulated drugs of the formulation matrix. In the present work, a difference spectrophotometric method is described for simultaneous determination of NF and TZ in the presence of each other in pure form and in pharmaceutical dosage forms (40).

## EXPERIMENTAL

### Apparatus

A Shimadzu (Kyoto, Japan) UV-1601 PC, UV-Visible double-beam spectrophotometer with two matched 1 cm path-length quartz cells. The subsequent statistical manipulation was performed by transferring the spectral data to Microsoft Excel 2007 program and processing them with the standard curve fit package and matrix calculations.

### Materials and reagents

All reagents and solvents were of analytical grade. NF pure sample was kindly supplied by Egyptian International Pharmaceutical Industries Company (EIPICO), Cairo, Egypt. TZ pure sample was kindly supplied by Medical Union Pharmaceuticals, Cairo, Egypt. 0.1 N Hydrochloric acid and sodium hydroxide were prepared.

### Formulations

Conaz^®^ tablets (Pharaonia Pharmaceuticals for Wockhardt, Egypt) batch number 916 9019 labeled to contain 400 mg NF and 600 mg TZ/tablet, were obtained from the local pharmacy.

### Solutions

Stock solutions of the two drugs were prepared separately by transferring 10 mg and 50 mg of NF and TZ respectively into two different 50 mL volumetric flasks; about 40 mL methanol was added to each flask (and sonicated at 37°C for about 5 min for NF only). The flasks were made up to the volume with the same solvent to obtain final concentration of 0.2 and 1 mg/mL of NF and TZ, respectively.

Stock solution of the two drugs was prepared simultaneously by transferring 10 and 15 mg of NF and TZ, respectively into 50 mL volumetric flask; about 40 mL methanol was added and sonicated at 37°C for about 5 min. The flask was made up to the volume with the same solvent to obtain final concentration of 0.2 and 0.3 mg/mL of NF and TZ, respectively.

The prepared solutions were protected from light throughout the study.

## GENERAL PROCEDURE

### Construction of Calibration Curves

Accurately measured aliquots equivalent to 20-200 μg NF and 50-500 μg TZ were quantitatively transferred from the stock solutions of the two drugs into two separate sets of 10 mL volumetric flasks. The set of each drug consists of two series. The first series of flasks were made up to the volume with 0.1 N NaOH and the absorption spectra were recorded against blank solution of 0.1 N NaOH. The flasks of the second series were made up to the volume with 0.1 N HCl and the absorption spectra were recorded against blank solution of 0.1 N HCl. The absorbance difference (δ A) between acidic solution and equimolar basic solution were measured by subtracting the spectra of the second series (in 0.1 N HCl) from the spectra of the first one (in 0.1 N NaOH) for each concentration of both drugs. The absorbance of the solutions of pure NF and TZ were taken immediately, zero time after preparation. The (δ*A*) values of the difference absorption spectra at 291.6 nm for NF and 344.4 nm for TZ were plotted *vs.* concentration of each drug (μg/mL) to get the calibration graphs. Alternatively, the corresponding regression equations were derived.

### Determination of NF and TZ in synthetic mixture

Aliquots from stock solution of the two mixed drugs were transferred into a series of three 10 mL volumetric flasks to prepare three samples of equimolar solution of NF and TZ in 0.1 N HCl and 0.1 N NaOH containing 6:9, 8:12, 10:15 μg/mL of NF and TZ, respectively to compare their results with the test solution in order to evaluate the specificity of the method for samples containing different concentrations of NF and TZ.

### Determination of NF and TZ in Conaz^®^ tablets

Twenty Conaz^®^ tablets were accurately weighed and pulverized. A quantity of the thoroughly mixed powder equivalent to 50 mg NF and 75 mg TZ was transferred into a 250 mL volumetric flask, about 230 mL methanol was added. The solution was sonicated and heated for 60 min at 48°C. Then the solution was made up to the volume with methanol. Then procedures of synthetic mixture were applied to determine NF and TZ in Conaz^®^ tablets. The content of each drug was determined by triplicate measurement of three independently prepared solutions. The nominal contents were determined either from the previously plotted calibration graphs or using the corresponding regression equations.

### Method validation

The developed method was validated according to ICH guidelines (41). The following parameters were considered: specificity, linearity, limit of detection (LOD), limit of quantitation (LOQ), accuracy, and precision.

### Specificity

The proposed method was successfully applied to tablets without interference from the excipients.

### Linearity

The method is linear over the range of 2-20 and 5-50 μg/mL for NF and TZ respectively. Linear regression equations were obtained. The regression plots in Table 1 showed a linear dependence of δ A values on drug concentration over the selected range. The linearity of calibration graphs and adherence to Beer’s law were validated by the high value of the correlation coefficient.

**Table 1:** Analytical data for the simultaneous determination of NF and TZ adopting the proposed method

Parameters	NF	TZ

Concentration range (μg/mL)	2-20	5-50
Correlation coefficient (r)	0.9999	0.9999
Slope	-0.0378	0.0149
Intercept	0.0089	0.0093
% RSD	0.93	0.74
% Er	0.29	0.23
S_y/x_	3.84 × 10^-3^	2.34 × 10^-3^
S_a_	2.62 × 10^-3^	1.60 × 10^-3^
S_b_	2.11 × 10^-4^	5.16 × 10^-5^
LOD (μg/mL)	0.23	0.36
LOQ (μg/mL)	0.70	1.08

LOD, limit of detection; LOQ, Limit of quantification; S_y/x_, standard deviation of the residuals; S_a_, standard deviation of the intercept; S_b_, standard deviation of the slope; % RSD, relative standard deviation (% RSD=SD × 100/X where SD is the standard deviation and X is the mean recovery); % Er, percent error (% Er=RSD/√n where n is the number of values).

### Limit of quantification (LOQ) and limit of detection (LOD)

The (LOQ) was determined according to ICH recommendations (41), to establish the lowest concentration that can be measured, below which the calibration graph is non linear (LOQ=10 σ/S where S is the slope and σ is the standard deviation of the intercept of regression line of the calibration curve). The (LOD) was determined by evaluating the lowest concentration of the analyte that can be detected (LOD=3.3 σ/S). The results of LOQ and LOD of NF and TZ by the proposed method were given in Table 1. The proposed method is sensitive as integrated by the high molar absorbtivity values of NF and TZ.

### Accuracy and Precision

Accuracy was calculated by % recovery of pure samples of intact drug and laboratory prepared mixtures of NF and TZ analyzed by the proposed methods (Table 2 & 3). Inter-day and intra-day accuracy and precision of the proposed method were also determined (Table 4).

The inter-day and intra-day precisions were examined by analysis of NF in concentrations 6, 8 and 10 μg/mL and TZ in concentrations 9, 12 and 15 μg/mL, each three times a day for three consecutive days. The inter-day and intra-day accuracy were proved by the low values of % Er. The relative standard deviations (RSD) for the results did not exceed 1.5%, proving the high reproducibility of the results and the precision of the method. This good level of precision was suitable for quality control analysis of NF and TZ in pharmaceutical dosage forms.

**Table 2:** Application of the proposed method and comparison method for the determination of NF and TZ in pure state

Drug determined	Proposed method					Reference method (34, 38)	
	Amount taken (μg/mL)	Amount found (μg/mL)	% Found	% RSD	ΔЄ (Mole^-1^. L^-1^)	Amount taken (μg/mL)	% Found

	2	1.99	99.50	0.85	10538.22	4	100.25
	4	3.93	98.25	0.84	11097.07	6	98.83
	6	6.07	101.17	0.94	11709.24	8	101.63
	8	7.93	99.13	0.79	11576.08	10	99.00
	10	10.06	100.6	0.96	11815.58	12	100.17
	12	11.99	99.92	0.99	11789.07		
	14	13.98	99.86	0.80	11815.58		
	16	16.19	101.19	0.88	11995.37		
	18	18.02	100.11	0.89	11886.47		
	20	19.85	99.25	0.94	11799.61		
	**% R ± SD = 99.90 ± 0.93**					**% R ± SD = 99.98 ± 1.13**	
	***t*-value = 0.19 (2.16)**						
	***F*-value = 1.49 (3.63)**						
	5	5.09	101.80	0.89	4203.59	6	100.00
	10	10.07	100.70	0.85	3931.62	9	99.33
	15	15.05	100.33	0.94	3840.88	12	100.58
	20	19.95	99.75	0.91	3783.26	15	100.13
	25	25.00	100.00	0.71	3768.43	18	99.78
	30	29.84	99.47	0.77	3733.81		
	35	34.68	99.09	0.86	3709.08		
	40	40.06	100.15	0.80	3739.99		
	45	45.04	100.09	0.78	3731.09		
	50	50.22	100.44	0.90	3738.75		
	**% R ± SD = 100.18 ± 0.74**					**% R ± SD = 99.96 ± 0.46**	
	***t*-value = 0.62 (2.16)**						
	***F*-value = 2.58 (6)**						

Figures between brackets are the tabulated t and F-values at (*P*=0.05). ΔЄ is the molar absorbativity. % R is the mean value of percent recovery. % RSD is calculated using the results of three separate determinations for each concentration.

**Table 3:** Application of the proposed method and comparison method for the simultaneous determination of NF and TZ in synthetic mixture in the ratio of 2:3 respectively

Drug determined	Proposed method			Reference method (34, 38)	
	Amount taken (μg/mL)		% Found	Amount taken (μg/mL)	% Found
	NF	TZ			

Norfloxacin	6	9	100.00	6	98.75
	8	12	100.50	8	100.28
	10	15	101.00	10	99.33
	**% R ± SD = 100.50 ± 0.50**			**% R ± SD = 99.45 ± 0.77**	
	***t*-value = 2.03 (2.78)**				
	***F*-value = 2.39 (19)**				
Tinidazole	6	9	99.50	9	99.39
	8	12	100.17	12	99.08
	10	15	99.87	15	100.12
	**% R ± SD = 99.85 ± 0.34**			**% R ± SD = 99.53 ± 0.53**	
	***t*-value = 1.64 (2.78)**				
	***F*-value = 2.53 (19)**				

Figures between brackets are the tabulated t and F-values at (*P*=0.05). In each of proposed and reference method, three samples of NF and TZ synthetic mixture were repeated.

## RESULTS AND DISCUSSION

In the present study, the difference absorption spectra of NF in 0.1 N NaOH *vs.* 0.1 N HCl showed zero crossing point at 344.4, 317 and 270 nm. The wavelength of 344.4 nm was chosen for measuring the absorbance of TZ, since the (δA) values of the TZ difference spectra at this point were more optimal and linear for accurate measurements of different concentrations of TZ. Similarly, the difference absorption spectra of TZ in 0.1 N NaOH *vs.* 0.1 N HCl showed zero crossing point at 291.6 and 242.5 nm, but the absorbance of NF was measured at the wavelength of 291.6 nm due to more linear (δA) values at this wavelength.

**Figure 2:**
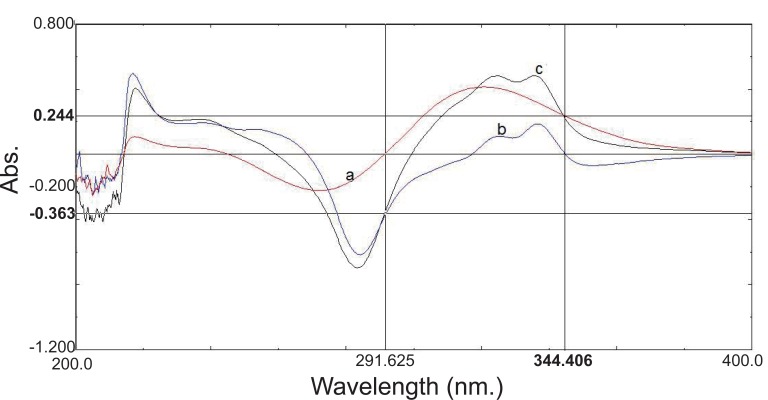
Difference absorption spectra of: a) TZ (15 μg/mL); b) NF (10 μg/mL); c) TZ (15 μg/mL) in presence of NF (10 μg/mL).

The method is based on alteration of the spectral properties of the two drugs using equimolar solutions of weak acid (0.1 N HCl) and weak base (0.1 N NaOH) then measurement of the absorbance difference (δA) between two solutions, provided the absorbances of the other absorbing interferants are not affected (Fig. 2).

### Optimization of experimental conditions

The optimum experimental conditions for the simultaneous determination of NF and TZ were established via a number of preliminary experiments. Such conditions were changed individually while others were kept constant. These conditions were listed as follows:

### Effect of time on the absorbance

Study of the time effect on the absorbance of NF and TZ was performed in 0.1 N NaOH *vs.* 0.1 N HCl. It was found that the absorbance of TZ increases gradually with the time, while the absorbance of NF is constant. Complete alkaline hydrolysis of TZ was accomplished upon heating for 5 min in 0.5 N NaOH as revealed by the constancy of the absorbance value at this condition after which no further increase in the absorbance value was obtained. It was clear that the use of 0.1 N NaOH without heating led to the gradual increase of TZ absorbance with time. However, in this study it was found that, measuring the absorbance of TZ instantaneously at zero time gave reproducible results with no need to wait for complete hydrolysis.

Addition of methanol to blank solution was not necessary, because the difference absorption spectrum of 1mL methanol (the maximum volume taken from stock solutions) in 0.1 N NaOH *vs.* 0.1 N HCl has no absorbance at 344.4 or 291.6 nm, where the absorbance was measured.

### Assay of synthetic mixture and dosage form

The proposed method was applied for simultaneous determination of NF with TZ in synthetic mixtures containing different concentrations of both drugs in ratio of (2:3) respectively. The δA values of standard solution of NF (0.2 μg/mL) and TZ (0.3 μg/mL) relative to the δ A values of the test solution was used for the determination of NF and TZ in the tablet preparations. The concentrations percent recoveries of both drugs in the synthetic mixture were calculated according to the corresponding regression equation. The results of synthetic mixture and dosage form are given in Table 3 & 5 respectively.

**Table 4:** Evaluation of the accuracy and precision data of the proposed method for the determination of NF nd TZ in Conaz tablet

Norfloxacin									
Amount added (μg/mL)	6.00		8.00		10.00	
	Amount found (μg/mL)	% Found	Amount found (μg/mL)	% Found	Amount found (μg/mL)	% Found

Intra-day	6.01	100.21	8.04	100.50	10.00	100.00
	6.00	100.00	8.01	100.13	9.92	99.20
	6.03	100.51	8.06	100.75	10.08	100.80
% R ± SD	100.24 ± 0.26		100.46 ± 0.31		100 ± 0.80	
% RSD	0.26		0.31		0.80	
% Er	0.15		0.18		0.46	
Inter-day	6.03	100.51	8.01	100.13	10.00	100.00
	6.04	100.66	7.94	99.27	9.95	99.50
	5.98	99.67	7.96	99.50	10.03	100.30
% R ± SD	100.28 ± 0.53		99.63 ± 0.45		99.93 ± 0.40	
% RSD	0.53		0.45		0.40	
% Er	0.31		0.26		0.23	
**Tinidazole**									
**Amount added (μg/mL)**	**9.00**		**12.00**		**15.00**	

Intra-day	8.99	99.89	11.95	99.58	15.05	100.33
	8.97	99.68	11.91	99.23	15.07	100.47
	8.93	99.22	11.97	99.79	14.96	99.73
% R ± SD	99.60 ± 0.34		99.53 ± 0.28		100.18 ± 0.39	
% RSD	0.34		0.28		0.39	
% Er	0.20		0.16		0.23	
Inter-day	8.99	99.89	11.97	99.79	15.05	100.33
	8.95	99.43	12.02	100.16	15.02	100.13
	9.02	100.17	12.04	100.35	14.98	99.87
% R ± SD	99.83 ± 0.37		100.1 ± 0.28		100.11 ± 0.23	
% RSD	0.37		0.28		0.23	
% Er	0.21		0.16		0.13	

Each result is the mean recovery of three separate determinations. Three samples of NF and TZ were repeated. Batch No. 916 9019.

**Table 5:** Assay results for the determination of NF and TZ in Conaz^®^ tablet by the proposed method and comparison method

Drug determined	Proposed method			Reference method (34, 38)
	Amount taken (μg/mL)	Amount found (μg/mL)	% Found	% Found

Norfloxacin	6	6.01	100.21	98.13
	8	7.96	99.50	101.21
	10	10.03	100.30	98.20
	**%R ± SD = 100.00 ± 0.44**			**%R ± SD = 99.18 ± 1.75**
	***t*-value = 1.95(2.78)**			
	***F*-value = 16.10 (19)**			
Tinidazole	9	9.02	100.17	101.43
	12	11.97	99.79	102.14
	15	14.98	99.87	101.35
	**% R ± SD = 99.94 ± 0.20**			**% R ± SD = 101.64 ± 0.43**
	***t*-value = 2.42 (2.78)**			
	***F-*value = 4.73 (19)**			

Figures between brackets are the tabulated t and F-values at (*P*=0.05). Each result is the mean recovery of three separate determinations. In each of proposed and reference method, three samples were repeated. Batch No. 916 9019.

## CONCLUSION

The present study described a validated difference spectrophotometric method for the simultaneous determination of NF and TZ in binary mixture. This technique enables the determination of either drug in the presence of the other by applying the zero-crossing technique in the spectra without prior separation steps. The proposed method can be easily applied as it does not require elaborate treatment of the samples and/or tedious procedures for extraction of the drugs. Furthermore, the method does not require expensive instruments and/or critical analytical reagents. The proposed method is sensitive as integrated by the high molar absorbtivity values of NF and TZ. The results obtained by the proposed methods are statistically compared with those obtained by the official one (Spectrophotometric derivative-ratio for NF and TZ) using Student’s t-test and variance ratio F-test from which it was concluded that there is no significant difference between them These advantages allowed the application of the proposed method to the analysis of pharmaceutical dosage forms containing mixture of NF and TZ in quality control laboratories.
